# Changes in cytomegalovirus load in the breast milk of very/extremely premature infants and the effect of pasteurization and freeze–thawing on reducing viral load

**DOI:** 10.3389/fped.2022.900249

**Published:** 2022-08-23

**Authors:** Tingting Huang, Wenhong Cai, Chen Ni, Shuhua Lai, Shuidi Lin, Quangui Wang

**Affiliations:** ^1^College of Clinical Medicine for Obstetrics and Gynecology and Pediatrics, Fujian Maternity and Child Health Hospital, Fujian Medical University, Fuzhou, China; ^2^Department of Neonatology, Fujian Provincial Maternity and Child Hospital, Fuzhou, China; ^3^Department of Clinical Laboratory, Fujian Provincial Maternity and Child Hospital, Fuzhou, China; ^4^Department of Pediatrics, Pingtan Comprehensive Experimental Area Maternal and Child Health Care Hospital, Fuzhou, China

**Keywords:** CMV DNA, breast milk, very/extremely premature infants, pasteurization, freeze-thawing, CMV infection

## Abstract

**Objective:**

This study aimed to clarify the change in Cytomegalovirus (CMV) loads in breast milk (BM) of very/extremely premature infants (VPI/EPI) with birth weight < 1,500 g after birth, and to compare the effectiveness of pasteurization and freeze–thawing methods in reducing the CMV load of BM.

**Methods:**

Breast milk samples were collected and tested every 2 weeks by fluorescence quantitative polymerase chain reaction (FQ-PCR). We determined CMV load in BM before and after pasteurizing, and freeze-thawing.

**Results:**

Cytomegalovirus DNA can already be detected in colostrum. The viral load gradually increased in the first 4 weeks, peaked in the 4th to 6th weeks, and gradually decreased thereafter. The viral load gradually returned to the initial level approximately 10–12 weeks postpartum. During the peak period of the CMV load in BM, the viral load was higher in the EPI than the VPI (*P* < 0.05). The average CMV load (logarithmic [LG]) in the pasteurization group was significantly lower than that in the raw BM group. The average CMV load in the freeze-thawed BM group was significantly lower than that in the raw BM group. The mean CMV load in the pasteurized BM group was lower than that in the freeze–thawed BM group, but the difference was not statistically significant. The CMV-DNA clearance rate in pasteurized was higher than in freeze–thawed (*P* < 0.05).

**Conclusion:**

The CMV detoxification rate in BM is high and the peak load period is mainly between 4 and 6 weeks. The CMV load values detected are higher than the threshold values (7 × 10^3^ copy number/mL) of CMV infection that are reported in the literature as a concern. Both the freeze-thaw and pasteurization techniques can effectively reduce the CMV load.

## Introduction

The Cytomegalovirus-immunoglobulin G (CMV-IgG) positivity rate in the serum of women of childbearing age in China is as high as 90% (1, 2). After delivery, CMV can be reactivated in the mammary gland. Preterm infants, especially very/extremely premature infants (VPI/EPI), with birth weight < 1,500 g, lack maternal antibodies obtained through the placenta, and their immune function are immature. Breastfeeding has become the main cause of acquired CMV infection, which may cause multiple organ damage, and even death in severe cases (3).

Breast milk (BM) provides the optimal diet for newborns, particularly those born prematurely. However, how to breastfeed VPI/EPI with CMV DNA positive BM remains a point of contention. There have been only limited investigations on the rules of CMV detoxification in VPI/EPI, and there is still contention regarding the most effective therapies for CMV DNA-positive BM. The CMV load level in the breast milk of VPI/EPI was evaluated at different times after birth in this study to better understand the CMV infection features and apply that knowledge to the CMV detoxifying practices for BM. A strategy to reduce CMV load in breast milk was discovered by comparing the reduction of CMV load in breast milk following pasteurization and freeze-thaw treatment.

## Methods

### Research object and grouping

The inclusion criteria of the study were as follows: newborns hospitalized in the neonatal department of our hospital from January 2020 to December 2020 who met the following three requirements: (1) hospitalized within 24 h after birth; (2) VPI/EPI with birth weight < 1,500 g (gestational age at birth 28–32 weeks/gestational age at birth < 28 weeks); and (3) breastfeeding during hospitalization. The exclusion criteria were as follows: (1) congenital abnormalities or malformations; (2) death occurring before the study was completed; and (3) failure to submit BM for examination according to study design specifications.

The Experimental Group comprised nursing mothers of VPI/EPI included in the study. We collected BM every 2 weeks until 12 weeks postpartum or discharge. Overall, 100 CMV DNA-positive milk samples were collected at 2, 4, 6, 8, and 10 weeks postpartum, with 20 samples in each period. The collected milk was equally divided into three groups: the fresh BM group, the pasteurization group, and the freeze-thaw treatment group.

This study was approved by the Ethics Committee of the Fujian Provincial Maternity and Child Hospital. Informed consent was obtained from the parents of the children.

### Collection and treatment of specimens

Breast milk was collected from very/extremely preterm mothers enrolled in the study at 0–5 days, 2, 4, 6, 8, 10, and 12 weeks after birth, or until discharge. Each time, 3–15 mL of BM was collected and evenly divided into three disposable sterile test tubes.

In the Raw BM group, milk samples were stored at 4°C. In the pasteurized BM groups, the samples were heated in a 62.5°C water bath for 30 s. In the freeze–thawed BM groups, the samples were frozen at −20°C for 72 h, melted naturally at 4°C, and then rewarmed in a warm water bath at 40°C for 30 min.

### Outcome criteria

After the fluorescence quantitative polymerase chain reaction (FQ-PCR) instrument generated the curve, the specific quantitative value of CMV load was extracted from BM samples using the automated extraction system. The result was judged as negative when the quantitative value was <500 copies/mL, and positive when the quantitative value suggested by the BM sample was >500 copies/mL. Subsequently, the load of CMV DNA test values was obtained from the standard curve suggested by the system.

### Definition

The CMV clearance rate was defined as the number of BM samples that initially gave a positive CMV test result and then a negative result, divided by the total number of CMV-positive breast milk samples. For this calculation, a conversion was defined when the CMV load dropped below 500 copies/mL.

The CMV detoxification rate was defined as the number of BM samples with a positive CMV DNA test divided by the total number of BM samples.

### Statistical analyses

Statistical analyses were performed using SPSS 26 software. The CMV DNA load value was logarithmic (LG). The number of cases and the percentage of the two groups were expressed (*N*, %). Comparisons between groups were performed using the chi-square test or Fisher’s exact probability test. If the measurement data of the two groups met the normality and homogeneity of variance, the data were expressed as the mean ± standard deviation, and the *T*-test was adopted. If the measurement data of the two groups did not conform to normal distribution or uneven variance, the data were described by the median and quartile spacing [M(Q)], and the comparison between groups was performed using the Mann–Whitney *U* test. If the measurement data of the three groups met the standard of normality and homogeneity of variance, they were expressed as the mean ± standard deviation. One-way ANOVA was used, and SNK (Student–Newman–Keuls) was used for pial comparison between groups. If the measurement data of the three groups did not conform to normal distribution or uneven variance, they were expressed as median and quartile spacing [M(Q)], and the Kruskal–Wallis H test was used. The *P*-value represents the difference between the data sets, and *P* < 0.05 was considered statistically significant.

## Results

### Characteristics of the study population

**FIGURE 1:**
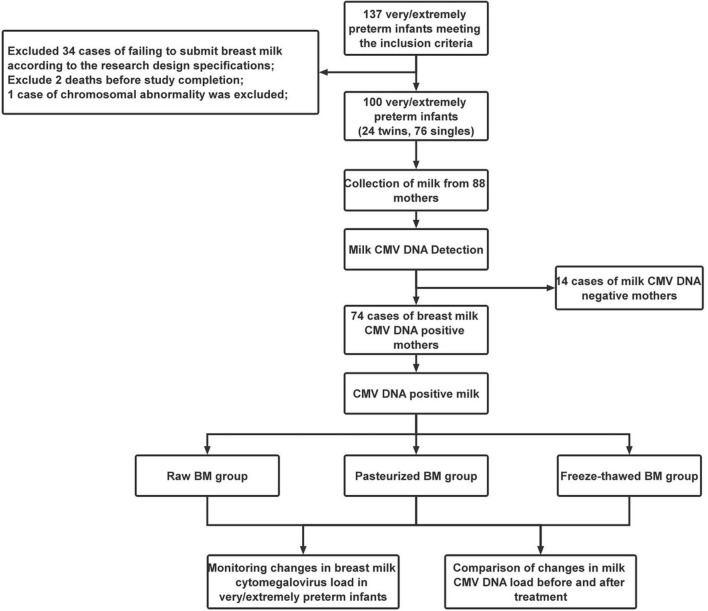
Flow Chart of the study.

Initially, 137 VPI/EPI who met the inclusion criteria were enrolled for this study, but 37 were subsequently excluded, including 34 cases of failure to submit BM according to study design specifications, two deaths before study completion, and one chromosomal abnormality. Finally, 100 premature infants from 88 different mothers were enrolled in the study. Of these, there were 24 cases of twin premature infants and 76 cases of single premature infants. Figure 1 shows the flow of the study.

### Cytomegalovirus transmission from breast milk

#### Cytomegalovirus DNA positivity rate in breast milk

In this study, we collected BM samples from 88 mothers. CMV DNA of BM was tested every 2 weeks until 12 weeks after delivery, or until discharge. The positivity rate of CMV DNA in BM was 84.1% (74/88), including a rate of 76.9% (10/13) in the BM of EPI and 85.3% (64/75) for VPI. There was no significant difference between the groups (*P* > 0.05, Table 1).

**TABLE 1:** Comparison of positive rates of cytomegalovirus (CMV) DNA in very/extremely premature infants (VPI/EPI) breast milk (BM).

	<28 week *N* = 13	28–32 week *N* = 75	χ^2^	*P*
Total positive rate (*N*, %)	10 (76.9)	64 (85.3)	–^b^	0.427
Colostrum positive rate (*N*, %)	2 (15.4)	16 (21.3)	–^b^	1.000

^b^Applies Fisher’s exact probability method.

#### Variation in cytomegalovirus load in breast milk

**FIGURE 2:**
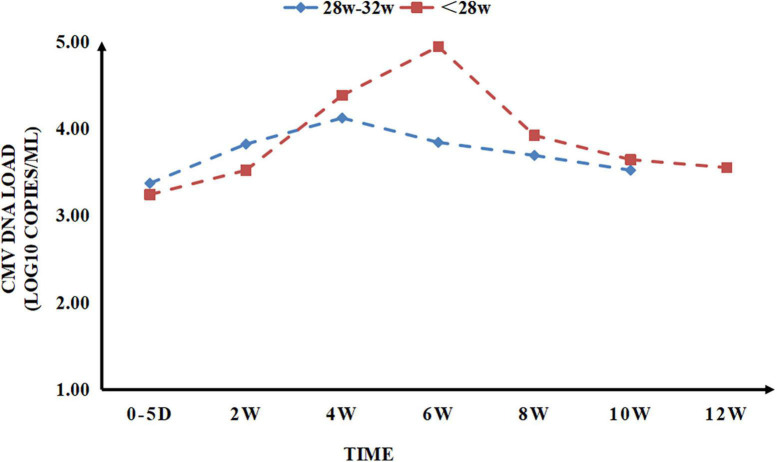
Cytomegalovirus (CMV) detoxification rule of very/extremely premature infants (VPI/EPI).

In our study, CMV DNA could already be detected in colostrum (0–5 days), and the earliest detection time was 3 days after delivery. CMV load showed a gradually increasing trend in the first 4 weeks and peaked at about 4–6 weeks, subsequently beginning to decline gradually and dropping to colostrum level about 10–12 weeks after delivery (Figure 2). With the data collected once every 2 weeks, a peak of CMV load emerged in the preterm infants: the EPI peaked at week 6, and the VPI peaked at week 4. At the peaked CMV load in preterm infants, the CMV load in EPI was higher than that in VPI, and the difference was statistically significant (*P* < 0.05, Table 2).

**TABLE 2:** Comparison of cytomegalovirus (CMV) load in breast milk during peak detoxification periods.

	<28 week	28–32 week	*t*	*P*
	*N* = 13	*N* = 75		
Breast milk CMV DNA load (Log_10_ copies/mL)	4.94 ± 0.52	4.12 ± 0.68	2.842	0.006

#### Changes in cytomegalovirus load in breast milk after different treatments

One hundred BM samples were collected from 88 very/extremely preterm mothers. We collected 20 samples in each postpartum period (2, 4, 6, 8, and 10 weeks). The average load value of CMV in the Raw BM group was 1.1 × 10^4^ copies/mL, that in the pasteurized BM group was 3.5 × 10^3^ copies/mL, and that in the freeze–thawed BM group was 4.9 × 10^3^ copies/mL. We used the logarithm (LG) for the numerical CMV viral load. The average CMV load (LG) in the pasteurization group was significantly lower than that in the raw BM group (*P* < 0.05). The average CMV load (LG) in the freeze–thawed BM group was significantly lower than that in the raw BM group (*P* < 0.05). The mean CMV load (LG) in the pasteurized BM group was lower than that in the freeze–thawed BM group, but the difference was not statistically significant (Table 3).

**TABLE 3:** Comparison of cytomegalovirus (CMV) DNA load before and after breast milk treatment.

Group	*N*	CMV DNA load (Log_10_ copies/mL)	*F*	*P*
Raw BM	100	4.06 ± 0.67*^a^^b^	13.447	0.000003
Pasteurized BM	100	3.55 ± 0.78		
Freeze-thawed BM	100	3.69 ± 0.71		

P-value: one-way ANOVA; SNK-q test was used for pairwise comparison.

*^a^The raw BM group compared with the pasteurized BM group, P < 0.05.

*^b^The raw BM group compared with the freeze–thawed BM group, P < 0.05.

The CMV DNA negative conversion rate in the pasteurized BM group was 16% (16/100), and the CMV DNA negative conversion rate in the freeze–thawed BM group was 7% (7/100). The difference between the two groups was statistically significant (*P* < 0.05; Table 4).

**TABLE 4:** Cytomegalovirus (CMV) DNA clearance rate after pasteurization and freeze–thawing of breast milk (BM).

	Pasteurization *N* = 100	Freezing-thawing *N* = 100	χ^2^	*P*
CMV DNA clearance rate (*N*, %)	16 (16.0)	7 (7.0)	3.979	0.046

## Discussion

### Cytomegalovirus transmission in breast milk

The positivity rate of CMV-IgG in Chinese women has been found to be over 90% (1, 2). CMV can be locally reactivated and shed by the infected maternal mammary gland (4, 5). Studies have found that the positivity rate of CMV in BM fluctuates between 56.1 and 96% (6–9). In this study, the positivity rate of CMV DNA in the BM of VPI/EPI was 84.1%, which is quite high. There was no difference in the positivity rate of CMV DNA in BM between VPI and EPI.

Detoxification of CMV in BM generally begins mainly at 10 days postpartum. The viral load increased gradually with age, reaching a peak at approximately 4–8 weeks, and decreasing significantly at 9–12 weeks (5, 10–12). In this study, the positive rate of CMV DNA detected in the colostrum of VPI/EPI was 20.1%, which first appeared on the 3rd day after delivery. The CMV load gradually increased in the 4th week, peaked at approximately 4–6 weeks, and then gradually decreased to the colostrum level at approximately 10–12 weeks postpartum. This suggests that the CMV detoxification rules of BM in VPI/EPI can guide the choice of the timing of BM treatment.

Jim et al. (9), Wang et al. (13) showed that breastfeeding with high CMV load and prolonged BM CMV detoxification increased the incidence of CMV infection acquired from BM in premature infants. Domestic studies have found that CMV infection caused by BM transmission is more likely to occur when the BM CMV load is greater than 2.6 × 10^3^ copy number/mL (13). Some scholars also believe that the risk of CMV infection is higher for BM CMV load > 7 × 10^3^ copy number/mL, and the peak period for BM detoxification, that is, when the risk of CMV infection may be increasing, is at 4–8 weeks postpartum (1). In this study, the peak CMV load in BM appeared at week 6 in VPI and at week 4 in EPI. At the peak CMV load in BM, the viral load level of EPI was significantly higher than that of VPIs and higher than the threshold value of CMV load proposed in domestic studies (1, 13). This suggests that VPI/EPI are at risk of acquiring CMV infection through breastfeeding, and EPI are at higher risk than VPI.

### Effect of pasteurization and freeze-thawing of breast milk on cytomegalovirus load

Currently, the commonly used methods for inactivating BM CMV mainly include freeze–thawing and pasteurization (3, 4, 14). Chiavarini et al. (8) and Hosseini et al. (15) showed that both pasteurization and freeze–thawing effectively reduced the CMV load levels in BM. Pasteurization includes long pasteurization (62.5°C for 30 min) and high-temperature pasteurization (72°C for 5–10 s). Long pasteurization, while effective at reducing the risk of CMV infection, results in the loss of some nutritional and immune components in BM. High-temperature pasteurization has little effect on the active ingredients in BM, but it is not easy to control clinically. Therefore, long pasteurization is still the most commonly used method of BM disinfection recommended by international BM banks (16). Freeze-thawing of BM is also considered to be effective at reducing CMV load levels while avoiding the impact on BM composition. However, other studies have suggested that freeze–thawing cannot reduce CMV load (17).

In a study, CMV infectivity was quantified using sensitive detection techniques like CMV late mRNA and high-speed centrifugal microculture assays. The results confirmed that late mRNA and viral infectivity were eliminated after pasteurization but not after freeze-thawing (18). Furthermore, the results showed that both techniques can effectively reduce the CMV load level in BM. The CMV load reduction and clearance rates of pasteurized BM are higher than those of freeze–thawed BM. There was still CMV DNA in pasteurized and freeze–thawed BM, and the clearance rate of CMV DNA was not high. Therefore, the evaluation of the viral infectivity of CMV requires further laboratory examination and the support of clinical trial data.

## Conclusion

The BM CMV detoxification rate is high in VPI/EPI. During the peak of the BM CMV load, the viral load level was significantly higher for EPI than that of the VPI, and both were significantly higher than the threshold value of viral load leading to CMV infection. Therefore, VPI/EPI are both at risk of acquiring CMV infection through breastfeeding. Both pasteurization and freeze–thawing can effectively reduce the CMV load of BM. The CMV clearance rate of pasteurized BM is higher. VPI and EPI have different BM CMV load peak periods. It is recommended to adopt an individualized breastfeeding strategy in preterm infants according to the change in BM CMV load during the first months after birth.

## Data availability statement

The raw data supporting the conclusions of this article will be made available by the authors, without undue reservation.

## Ethics statement

The studies involving human participants were reviewed and approved by the Ethics Committee of the Fujian Provincial Maternity and Child Hospital. Written informed consent to participate in this study was provided by the participants’ legal guardian/next of kin.

## Author contributions

WC and TH designed the study and submitted the manuscript. TH and CN collected the data. SLa and QW analyzed the data together. TH and SLi drafted the manuscript. WC supervised this study. All authors read the final version of this article and approved the submitted version.
